# Hair Growth-Promoting Effect of *Hydrangea serrata* (Thunb.) Ser. Extract and Its Active Component Hydrangenol: In Vitro and In Vivo Study

**DOI:** 10.3390/ijms251910370

**Published:** 2024-09-26

**Authors:** Soyoon Park, Hyunjae Kim, Hye Shin Ahn, Changseon Na, Yu-Kyong Shin

**Affiliations:** Department of New Material Development, COSMAXBIO, Seongnam 13486, Republic of Korea; soyoon.park@cosmax.com (S.P.); hyounjeakim@cosmax.com (H.K.); hsahn@cosmax.com (H.S.A.); csna@cosmax.com (C.N.)

**Keywords:** *Hydrangea serrata* (Thunb.) Ser., hydrangenol, hair growth, growth factors, anti-inflammation, anti-androgen, dermal papilla cells, C57BL/6 mice, 3D human skin organoid

## Abstract

With the escalating prevalence of hair loss, the demand for effective hair loss treatment has surged. This study evaluated the effects of hot water extract of *Hydrangea serrata* (Thunb.) Ser. leaf (WHS) on hair growth, employing cell cultures, mice, and human skin organoid models. Both WHS and hydrangenol were found to enhance 5α-reductase inhibitory activity. WHS and hydrangenol have been shown to stimulate dermal papilla cell (DPC) growth, potentially through factors like keratinocyte growth factor (*KGF*), fibroblast growth factor 10 (*FGF10*), and transforming growth factor-β1 (*TGF-β1*). They also elevated the expression levels of keratin genes (*K31* and *K85*) and the ceramide synthase (*CerS3*) gene, crucial clinical indicators of hair health. Furthermore, they exhibited notable anti-inflammatory and anti-androgenic properties by reducing the levels of tumor necrosis factor-α (*TNF-α*) and androgen signaling molecules, including androgen receptor (*AR*) and dickkopf-1 (*DKK-1*) gene expression. Oral administration of WHS to C57BL/6 mice for 3 weeks confirmed its hair growth-promoting effects, improving hair growth parameters and gene expression without significant changes in hair weight. Additionally, in a human skin organoid model, WHS was found to stimulate hair formation and augment the expression of follicle markers. These findings position WHS as a promising nutraceutical for promoting hair health, as evidenced by its efficacy in both in vitro and in vivo models.

## 1. Introduction

Hair grows from hair follicles embedded in the skin, with the follicle size being a key factor in determining hair shaft diameter [1,2,3]. Unfortunately, hormone imbalances, excessive chemical usage, and psychological stress can cause hair follicles to shrink, resulting in fragile, easily breakable hair and eventually leading to hair loss [4]. Among the various types of cells in hair follicles, dermal papilla cells, located at the base of the hair follicles, play a crucial role in regulating the size of the hair follicles by secreting growth factors. An increase in the secretion of growth factors by dermal papilla cells leads to their growth and eventually enhances the diameter of hair follicles [1,5]. Therefore, promoting the growth of dermal papilla cells should be a key strategy in preventing hair loss.

Another important strategy for alleviating hair loss is to suppress the factors that contribute to the condition, particularly those that generate reactive oxygen species (ROS). ROS are highly reactive molecules that can damage cellular structures, including membrane lipids, proteins, and DNA. Oxidative stress, resulting from an imbalance between ROS and antioxidant defenses, is known to be associated with the senescence of DPCs [6,7,8]. Androgens, especially testosterone, play a major internal role in this process; testosterone is converted by 5α-reductase into 5α-dihydrotestosterone (DHT), a more biologically active form that inhibits DPC proliferation and suppresses hair growth [9,10]. Among the treatments for hair loss approved by the Food and Drug Administration (FDA), finasteride acts as an inhibitor of 5α-reductase, which is a significant contributor to hair loss. However, the development of safe natural compounds is urgently needed due to the various side effects like mood disturbance, cognitive symptoms, and sexual dysfunction associated with this synthetic chemical [11,12].

To explore safe substances for preventing hair loss, screening was conducted on over 200 natural products commonly used for culinary or medicinal purposes, with the aim of assessing their potential to inhibit 5α-reductase. As a result, *Hydrangea serrata* (Thunb.) Ser. exhibited the most remarkable inhibitory efficacy. *Hydrangea serrata* (Thunb.) Ser. is composed of oval leaves and blue or pink flowers, and its leaves are consumed as tea from ancient times [13]. *Hydrangea serrata* (Thunb.) Ser. leaves’ extract contains a variety of compounds, including quercetin 3-O-β-xylopyranosyl (1→2)-β-D-glucopyranoside, kaempferol 3-O-β-xylopyranosyl (1→2)-β-D-glucopyranoside, 4-hydroxybenzaldehyde, 3*S*-phyllodulcin 8-glucoside, 3*R*-phyllodulcin 8-glucoside, hydrangenol, and phyllodulcin. In particular, hydrangenol, the active component of *Hydrangea serrata* (Thunb.) Ser. leaves’ extract, is associated with antioxidant, anti-inflammatory, antimicrobial, and anticancer effects [14,15,16,17,18]. Previous clinical trials have also demonstrated a reduction in body fat and skin health as benefits of the water extract of *Hydrangea serrata* (Thunb.) Ser. leaves, confirming its non-toxicity for human consumption [19,20]. However, the effects of the water extract of *Hydrangea serrata* (Thunb.) Ser. leaves on hair loss remain unexplored. Therefore, this study aims to elucidate the hair growth-promoting effects of the water extract of *Hydrangea serrata* (Thunb.) Ser. leaves through in vitro and in vivo experiments.

## 2. Results

### 2.1. Effect of WHS and an Active Compound, Hydrangenol, on 5α-Reductase Inhibitory Activity

**Figure 1:**
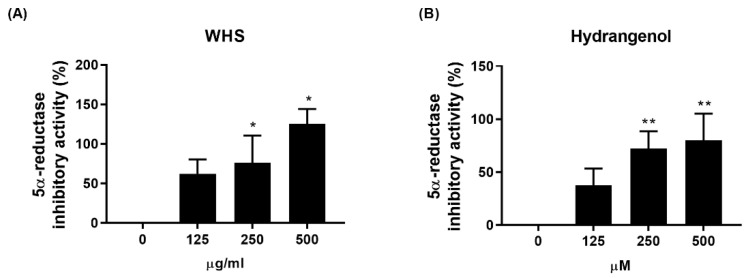
Inhibitory effects of WHS and hydrangenol on 5α-reductase activity in a cell-free system: (**A**,**B**) The activity of 5α-reductase was assessed using several concentrations. Data are expressed as mean ± standard error of the mean (SEM) of three experiments. Statistical significance is denoted as * *p* < 0.05, ** *p* < 0.01 compared with the control group. WHS; water extract of *Hydrangea serrata* leaves.

To explore the impact of WHS and hydrangenol, an active compound, on the inhibition of 5α-reductase activity, various doses of WHS (125, 250, and 500 μg/mL) or hydrangenol (125, 250, and 500 μM) were used. Notably, significant inhibition of 5α-reductase activity was observed at a dose of 250 μg/mL for WHS and 250 μM for hydrangenol, with the effect increasing in a dose-dependent manner (Figure 1A,B).

### 2.2. Effect of WHS and Hydrangenol on DPC Growth

**Figure 2:**
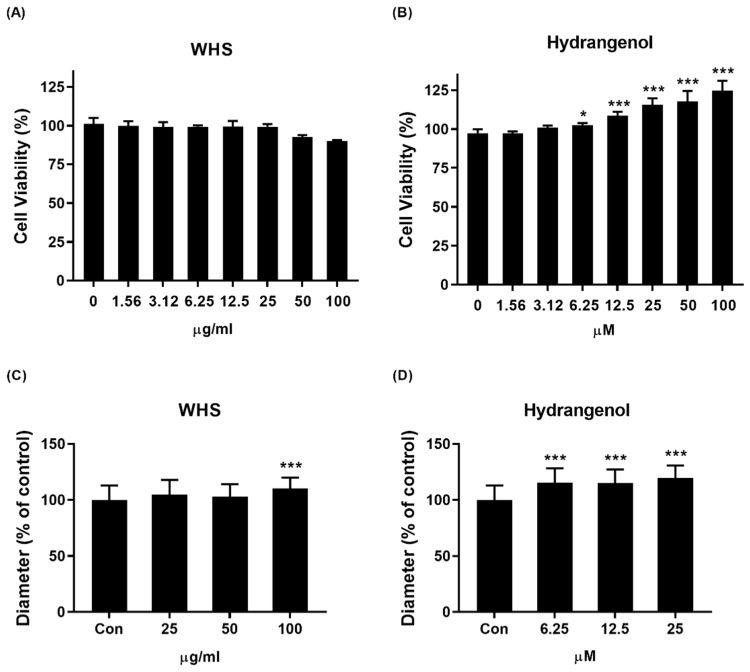
Effect of promoting DPC growth in response to WHS or hydrangenol treatment: (**A**,**B**) cell viability was determined following treatment with WHS or hydrangenol at indicated concentrations using MTT assay; (**C**,**D**) the size of DPC spheroids’ diameter was quantified using phase-contrast microscopy. Data are expressed as mean ± standard error of the mean (SEM) of three experiments. Statistical significance is denoted as * *p* < 0.05, *** *p* < 0.001 compared with the control group. WHS; water extract of Hydrangea serrata leaves.

To confirm the hair growth-promoting capacity in DPCs, we investigated whether WHS or hydrangenol can increase the size of the DPC spheroids. For cell viability evaluation, cells were treated with either vehicle control (DMSO) or seven different concentrations of WHS (1.56–100 μg/mL) or hydrangenol (1.56–100 μM) for 24 h. Both WHS and hydrangenol showed no toxicity to cells. Furthermore, hydrangenol increased DPC proliferation in a dose-dependent manner, and significant increases were observed after 6.25 μM (Figure 2A,B). Therefore, all further experiments were performed with 25–100 μg/mL of WHS or 6.25–25 μM of hydrangenol. For DPC spheroids’ size evaluation, the cells were treated with either vehicle control (DMSO) or various concentrations of WHS or hydrangenol for 24 h. We confirmed that both WHS and hydrangenol were able to increase the diameter of spheroids (Figure 2C,D). Photographs of the spheroids are presented in Supplementary Figure S1.

### 2.3. Effect of WHS and Hydrangenol on Expression of Hair Growth-Related Genes in DPCs

**Figure 3:**
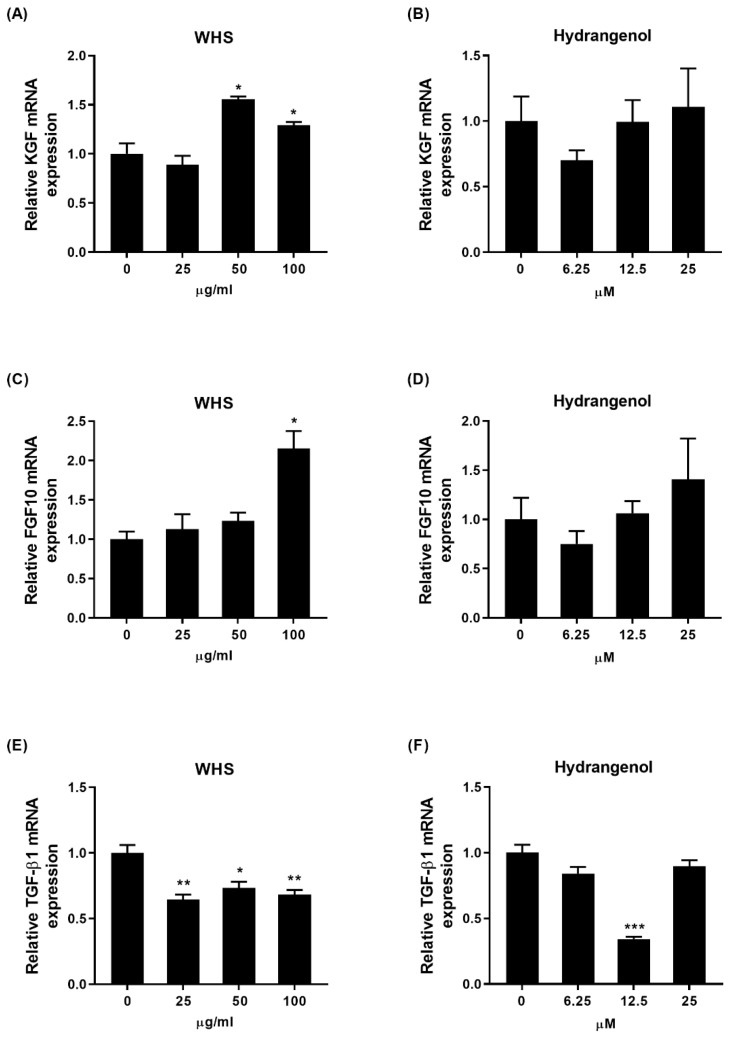
Changes in mRNA expression levels of growth-related genes by WHS and hydrangenol in DPCs. The mRNA expressions of (**A**,**B**) keratinocyte growth factor (KGF), (**C**,**D**) fibroblast growth factor 10 (FGF10), and (**E**,**F**) transforming growth factor-β1 (TGF-β1) were evaluated. Data are expressed as mean ± standard error of the mean (SEM) of three experiments. Statistical significance is denoted as * *p* < 0.05, ** *p* < 0.01, *** *p* < 0.001 compared with the control group. WHS; water extract of Hydrangea serrata leaves.

To unravel the mechanism of WHS and hydrangenol on hair growth, the DPCs were treated with WHS or hydrangenol for 24 h. Subsequently, the mRNA expression levels of growth-related genes, including keratinocyte growth factor (*KGF*), fibroblast growth factor 10 (*FGF10*), and transforming growth factor-β1 (*TGF-β1*), in the DPCs were then analyzed. KGF and FGF10 are known to stimulate hair growth, while TGF-β1 is a well-known factor that causes cell death, leading to hair loss. WHS significantly increased the expression levels of KGF and FGF10 while decreasing TGF-β1 levels (Figure 3A,C,E). Additionally, while the expression of KGF and FGF10 was not significantly altered in the hydrangenol-treated group, a decrease in TGF-β1 expression was observed (Figure 3B,D,F).

### 2.4. Effect of WHS and Hydrangenol on Expression of Keratin and Ceramide Synthase Genes in HaCaT

**Figure 4:**
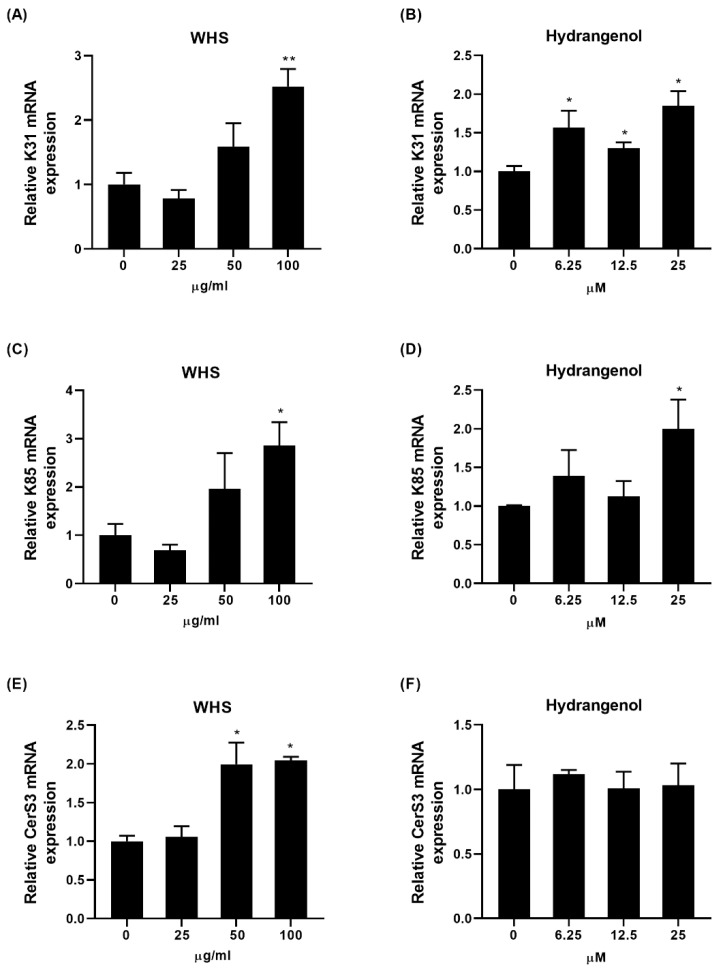
Changes in mRNA expression levels of keratin and ceramide synthase genes by WHS and hydrangenol in HaCaT. The mRNA expressions of (**A**,**B**) keratin 31 (K31), (**C**,**D**) keratin 85 (K85), and (**E**,**F**) ceramide synthase 3 (CerS3) were evaluated. Data are expressed as mean ± standard error of the mean (SEM) of three experiments. Statistical significance is denoted as * *p* < 0.05, ** *p* < 0.01 compared with the control group. WHS; water extract of Hydrangea serrata leaves.

To examine the effects on regulating the hair keratin and ceramide synthase expression, HaCaT keratinocytes were treated with WHS or hydrangenol for 24 h. Considering that keratin filaments are obligatory heteropolymers with opposite types, keratin 31 (*K31*; type I keratin) and keratin 85 (*K85*; type II keratin) were selected. WHS and hydrangenol both significantly upregulated K31 and K85 gene expression (Figure 4A–D). Additionally, as ceramide is a constituent of the hair cuticle barrier, the expression of the ceramide synthase 3 (*CerS3*) gene was assessed. While WHS significantly upregulated CerS3 gene expression, hydrangenol had no significant impact on it (Figure 4E,F).

### 2.5. Effect of WHS and Hydrangenol on Antioxidant Enzyme Activity and Expression of Inflammatory Gene in H_2_O_2_-Induced DPCs

**Figure 5:**
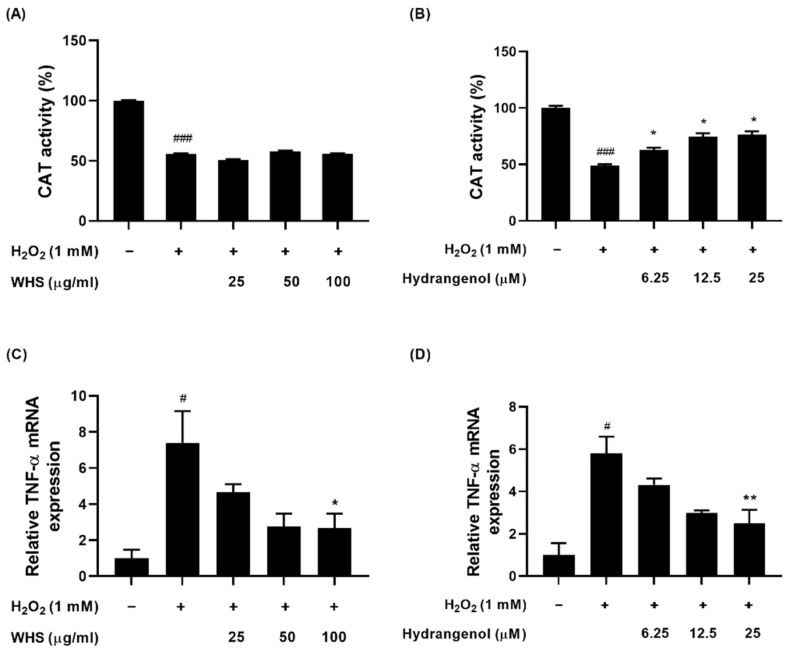
Antioxidant and anti-inflammatory activity of WHS and hydrangenol in H_2_O_2_-induced DPCs: (**A**,**B**) for antioxidant activity, catalase (CAT) activity was analyzed using a colorimetric assay; (**C**,**D**) tumor necrosis factor-α (TNF-α) was chosen as a biomarker for inflammation. The mRNA expression of TNF-α was analyzed. Data are expressed as mean ± standard error of the mean (SEM) of three experiments. Statistical significance is denoted as ^#^ *p* < 0.05, ^###^ *p* < 0.001 compared with the control group and * *p* < 0.05, ** *p* < 0.01 compared with the H_2_O_2_-treated group. WHS; water extract of Hydrangea serrata leaves.

To assess the antioxidant and anti-inflammatory effects of WHS and hydrangenol in H_2_O_2_-induced DPCs, the catalase activity and mRNA expression levels of inflammatory factors were measured. The H_2_O_2_-treated group demonstrated a significant reduction in catalase activity compared to the control. However, hydrangenol significantly enhanced catalase activity, while WHS showed no significant alteration in this regard (Figure 5A,B). As for inflammatory cytokines, tumor necrosis factor-α (*TNF-α*) is a prominent factor. The H_2_O_2_-treated group exhibited elevated expression levels of this inflammatory factor compared to the control. Both WHS and hydrangenol significantly lowered mRNA expression levels compared to the H_2_O_2_-treated group (Figure 5C,D).

### 2.6. Effect of WHS and Hydrangenol on Androgen Signaling Molecules in DHT-Induced DPCs

**Figure 6:**
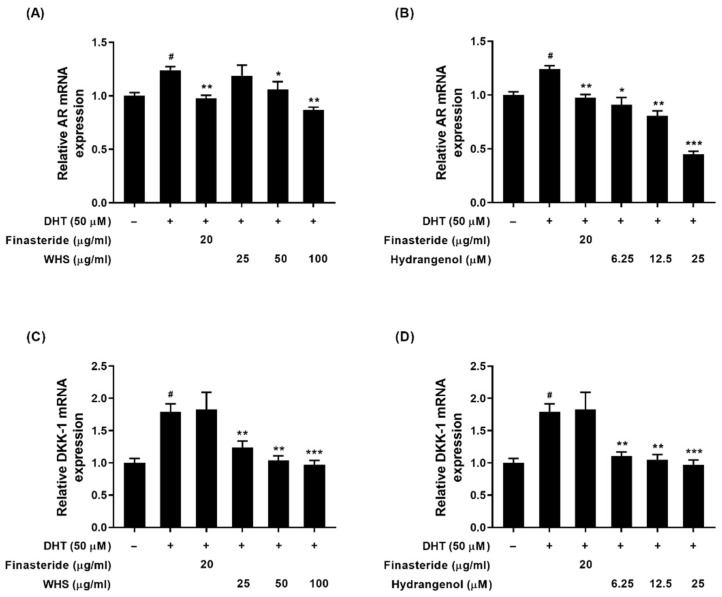
Anti-androgenic effects of WHS and hydrangenol in dihydrotestosterone (DHT)-stimulated DPCs. The mRNA expressions of (**A**,**B**) androgen receptor (AR) and (**C**,**D**) dickkopf-a (DKK-1) were measured. Finasteride was used as a standard drug. Data are expressed as mean ± standard error of the mean (SEM) of three experiments. Statistical significance is denoted as ^#^ *p* < 0.05 compared with the control group and * *p* < 0.05, ** *p* < 0.01, *** *p* < 0.001 compared with the DHT_-_treated group. WHS; water extract of Hydrangea serrata leaves.

To investigate the possible anti-androgenic effects of WHS and hydrangenol on DHT-induced DPCs, the mRNA expression levels of the androgen receptor (*AR*) and its downstream target, dickkopf-1 (*DKK-1*), were quantified. Finasteride was used as a positive control. The DHT-treated group showed a significant increase in AR and DKK-1 mRNA expression levels compared to the control. All treatments notably reduced the expression level of AR compared to the DHT-treated group (Figure 6A,B). Specifically, both WHS and hydrangenol groups exhibited lower expression levels compared to the finasteride group. Also, WHS and hydrangenol significantly decreased DKK-1 expression levels, while finasteride treatment did not exhibit significant changes (Figure 6C,D).

### 2.7. Effect of WHS on Hair Growth Promotion in C57BL/6 Mice

**Figure 7:**
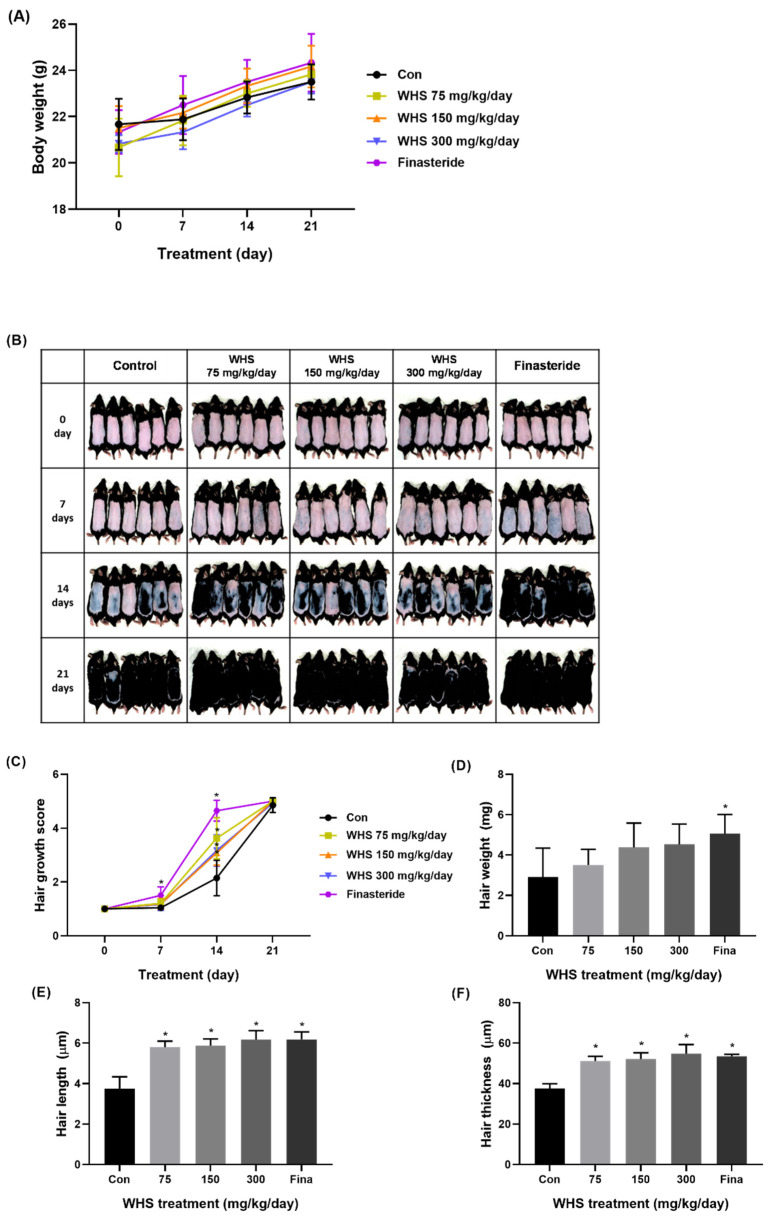
Hair growth-promoting effect of WHS in C57BL/6 mice. Mice were orally administered 75, 150, and 300 mg/kg/d doses of WHS once a day for 3 weeks: (**A**) body weight was measured during the experimental periods; (**B**) visual assessment of hair growth; (**C**) hair growth score was calculated; (**D**) hair weight, (**E**) length, and (**F**) thickness were measured. Data are expressed as mean ± standard error of the mean (SEM) of three experiments. Statistical significance is denoted as * *p* < 0.05 compared with the control group. WHS; water extract of Hydrangea serrata leaves.

**Figure 8:**
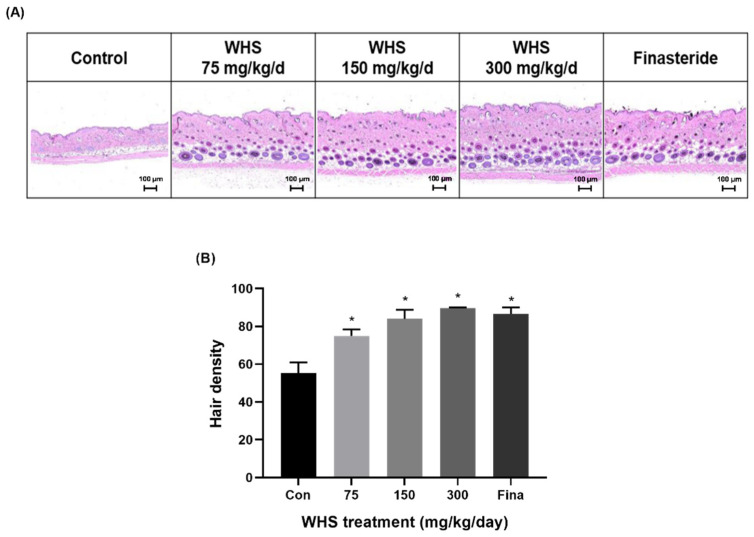
Hair-density-increasing effect of WHS in C57BL/6. The density of hair was evaluated after a 2-week administration of WHS: (**A**) histological analysis was performed by H&E staining; (**B**) the number of hair follicles in the epidermis and dermis tissues were counted. Data are expressed as mean ± standard error of the mean (SEM) of three experiments. Statistical significance is denoted as * *p* < 0.05 compared with the control group. WHS; water extract of Hydrangea serrata leaves.

To evaluate the potential promoting effect of oral WHS consumption on hair growth in vivo, WHS (75, 150, and 300 mg/kg/d) was orally administered to C57BL/6 mice daily for three consecutive weeks. Throughout the experimental periods, there was no difference in body weight among the groups (Figure 7A). For a visual assessment of hair growth in the mice, photographs were taken at 7-day intervals to observe the hair growth pattern on the dorsal skin. The visual evaluation indicated that the administration of WHS notably enhanced mouse hair growth compared to the control group. (Figure 7B). After three weeks of administration, WHS-treated mice exhibited significant increases in hair growth score, length, thickness, and density (Figure 7C,E,F and Figure 8A,B). Particularly, the hair growth-promoting effect of the WHS treatment was comparable to that of finasteride, confirming its efficacy in promoting hair growth. However, there were no significant differences observed in hair weight among the WHS group (Figure 7D).

### 2.8. Effect of WHS on Expression of Growth-Related Genes in C57BL/6 Mice

**Figure 9:**
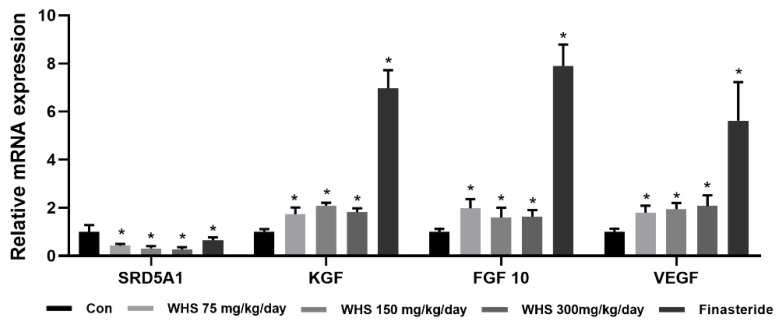
Changes in mRNA expression levels of growth-related genes by WHS in C57BL/6 mice. After 3 weeks of administration, the mRNA expressions of 5α-reductase 1 (SRD5A1), fibroblast growth factor 7 (FGF7), fibroblast growth factor 10 (FGF10), and VEGF were analyzed. Data are expressed as mean ± standard error of the mean (SEM) of three experiments. Statistical significance is denoted as * *p* < 0.05 compared with the control group. WHS; water extract of Hydrangea serrata leaves.

To determine the impact of WHS on the expression of growth-related genes in vivo, we analyzed the expression levels of 5α-reductase 1 (*SRD5A1*), fibroblast growth factor 7 (*FGF7*), fibroblast growth factor 10 (*FGF10*), and VEGF using RNA isolated from dorsal skin tissue of murine. The gene expression of SRD5A1 significantly decreased, while those of FGF7, FGF10, and VEGF significantly increased in the WHS group compared to the control group (Figure 9).

### 2.9. Effect of WHS on Enhancing Hair Formation in 3D Human Skin Organoids

**Figure 10:**
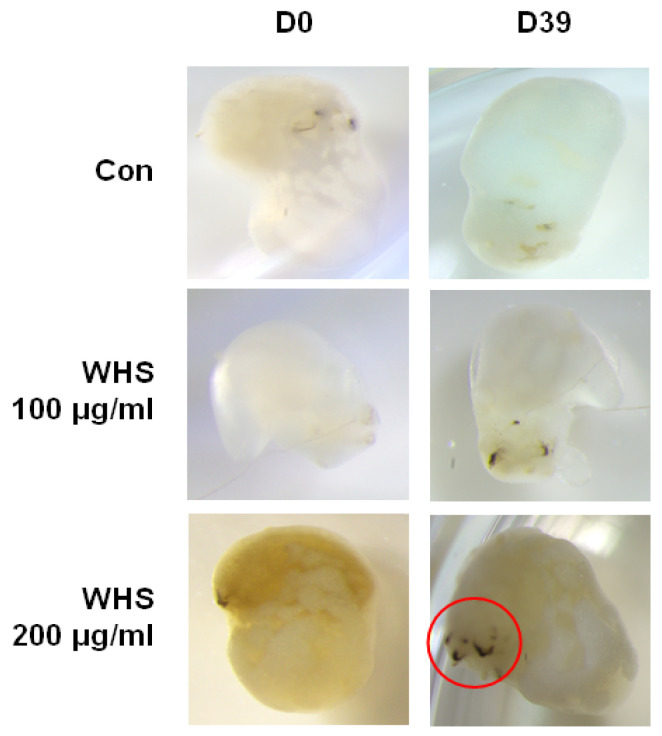
Effect of promoting hair formation by WHS in skin organoids. Hair formation was evaluated from day 131 to day 171 of WHS treatment. Representative images of organoids in response to WHS treatment at indicated concentrations were captured. The red circle indicates hair formation. WHS; water extract of Hydrangea serrata leaves.

To evaluate the ability of WHS to promote hair formation in a 3D human skin organoid model, cultured media were supplemented with 100 μg/mL or 200 μg/mL of WHS every three days, from day 131 to day 170. After 39 days of treatment, a visual assessment showed a marked increase in the WHS-treated group compared to the untreated conditions (Figure 10). Detailed data related to hair formation are presented in Supplementary Figures S2 and S3.

## 3. Discussion

As the hair loss population increases worldwide, various methods to improve hair loss are being studied. Currently, drugs approved by the FDA, such as minoxidil, finasteride, and baricitinib, are used to treat hair loss. However, due to the reported diverse side effects, there is growing interest in natural plant-based remedies. In most of the recent research, plant extracts such as *Gardenia florida*, *Mangifera indica*, and *Cucumis melo* have been highlighted for their potential impact on hair growth; however, the specific components responsible for such effects have not been reported [4,21,22]. In this study, for the first time, the effect of the hot water extract of *Hydrangea serrata* leaf (WHS) on hair loss improvement was investigated through in vitro (hDPCs, organoid) and in vivo (mice) experiments, confirming specific compounds responsible for these effects.

The size of hair follicles is closely related to the volume of the dermal papilla. Therefore, the growth of DPCs becomes a major indicator of hair regeneration and maintenance. In this study, WHS and the isolated main component, hydrangenol, both showed a significant increase in the diameter of the DPC spheroids, indicating enhanced cell proliferation and aggregation. Also, the activated DPCs significantly influence hair growth through changes in the surrounding environment. These environmental changes occur via growth factors secreted from the activated DPCs, acting in a paracrine manner and interacting with surrounding cells. For instance, KGF and FGF10 promote hair growth by stimulating cell proliferation in the hair matrix and hair root sheath, both of which originate from DPCs [23,24]. TGF-β1 inhibits the proliferation of hair matrix epithelial cells, thereby impairing the development of the hair shaft [25]. In this study, WHS increased the gene expression of KGF and FGF10 while decreasing TGF-β1, regulating growth factors favorably for hair growth. Therefore, the potential hair growth-promoting effect of WHS might be attributed to growth factors.

Another important clinical characteristic of hair health is its elasticity and glossiness. The structure of hair consists of three layers: the cuticle, cortex, and medulla [26]. The cuticle forms a protective barrier that plays a pivotal role in maintaining moisture and enhancing hair gloss [27,28]. The cortex, constituting the majority of hair volume, comprises keratin proteins arranged in a helical structure, significantly contributing to hair elasticity [27,29,30]. The medulla, located at the innermost part of the hair, supports the hair structure [27]. In our study, we measured the mRNA expression levels of K31, located throughout the entire cortex, and K85, found in the matrix, precortex, and cuticle, as indicators of hair elasticity [31,32]. Additionally, we examined mRNA expression levels of CerS3, responsible for barrier lipid ceramide synthesis, as indicators of hair glossiness [33,34]. Our findings revealed an increase in the expression levels of K31 and K85 genes following treatment with WHS and hydrangenol, as well as CerS3 gene expression following treatment with WHS, offering insights into their potential contributions to overall hair quality.

Oxidative stress has historically been considered one of the reasons behind hair loss [35]. The continued exposure of the scalp to intense UV rays or air pollutants leads to the accumulation of oxidative stressors like H_2_O_2_, inducing senescence in follicular cells and interrupting hair growth [35,36,37]. To counteract the acceleration of hair loss in response to environmental stress, we focused on evaluating the activity of endogenous antioxidant enzymes. Antioxidant enzymes such as superoxide dismutase (SOD), catalase (CAT), and glutathione peroxidase (GPx) play a crucial role in cellular defense against oxidative stress [38]. Among them, CAT is an important enzyme that decomposes H_2_O_2_ into water and oxygen, consequently curtailing H_2_O_2_-induced cell damage [39,40]. Additionally, there is a possible connection between oxidative stress and the increase in the secretion of proinflammatory cytokines such as TNF-α. Some researchers have reported that TNF-α can contribute to the condensation of the dermal papilla, ultimately leading to the destruction of hair follicle structure [41]. Although this study did not demonstrate antioxidant activity for WHS, it is plausible that the antioxidant properties of hydrangenol enhanced the anti-inflammatory effects of WHS. Previous research has shown that ROS activates the NF-κB p65 inflammatory response pathway in DPCs, leading to an increase in various cellular inflammatory factors, including TNF-α [42]. Another study revealed that CAT inhibits the generation of ROS from H_2_O_2_ [43]. Therefore, these findings suggest that the increase in CAT activity by hydrangenol suppresses ROS generation, contributing to the anti-inflammatory effects observed in WHS. Future research should verify whether the NF-κB p65 pathway is indeed inhibited by hydrangenol.

Given that androgenetic alopecia is prevalent among many hair loss patients, our study aimed to determine whether WHS inhibits the mechanism involving male hormones [44,45]. Previous human studies have consistently confirmed the negative influence of the Wnt mechanism in DPCs among individuals with androgenetic alopecia patients [46,47]. Consequently, the significant role of male hormones in causing hair loss might be due to their interaction with the Wnt mechanism, well known for its involvement in hair growth. As is known, an inhibitor of the Wnt mechanism, DKK-1, is a key factor in hair loss induction, ultimately suppressing follicular formation [48]. In this context, our results suggest that WHS could support hair growth by preventing the androgenic pathway through the downregulation of DKK-1 levels.

To conduct a more in-depth efficacy evaluation, we employed both a C57BL/6 mouse model and a 3D human skin model. C57BL/6 mice, known for having melanocytes specifically in hair follicles, exhibit melanogenesis correlated with the hair growth cycle, rendering them a suitable model for hair loss studies [49]. Although a mouse model provides a holistic perspective on physiological responses and systemic effects, it still has limitations in accurately representing these effects in the human body. Thus, to address disparities between animal and human physiology, we complemented our approach with experiments using a 3D human skin equivalent model [50]. In our study, we discovered that WHS induces hair growth and hair follicle development in the 3D organoid model.

In our analysis, we identified a diverse array of compounds within WHS. Among them, hydrangenol has been regarded as an active compound based on our previous studies, which reported its anti-photoaging effects both in vitro and in vivo [13,51]. Following a similar rationale, we hypothesized that hydrangenol might also promote hair growth, which was confirmed in this study. While other compounds have not been previously reported for their hair growth-promoting effects, we speculate that hydrangenol could synergize with them, potentially enhancing overall hair growth. Consequently, further investigation into their combined interactions or multifactorial effects on hair growth should be conducted.

Although our study used cell, mice, and human organoid models to explore the effects of WHS on hair health, we recognize certain limitations associated with our experimental methodology. The inherent complexities of human physiology, not fully recapitulated in experimental models, necessitate thorough clinical validation. Furthermore, while our findings demonstrate promising outcomes in controlled environments, the translation to diverse patient populations remains challenging. Given the rising demand for oral supplements targeting hair concerns, the finding holds significant potential, yet clinical trials are essential to establish efficacy, safety, and therapeutic relevance in clinical settings [52].

## 4. Materials and Methods

### 4.1. Reagents

WHS and hydrangenol were prepared as described previously [13,14], and the high-performance liquid chromatography (HPLC) profile is shown in Supplementary Figure S4. Dulbecco’s modified Eagle’s medium (DMEM), fetal bovine serum (FBS), and trypsin were obtained from HyClone (Logan, UT, USA), and antibiotic–antimycotic (AA) was purchased from GenDEPOT (Barker, TX, USA). The DPC growth medium with supplement mix was obtained from PromoCell (Heidelberg, Germany). Phosphate-buffered saline (PBS) was purchased from Welgene (Daegu, Republic of Korea), and 3-(4,5-dimethylthiazol-2-yl)-2,5-diphenyl tetrazolium bromide (MTT), dimethyl sulfoxide (DMSO), dihydrotestosterone (DHT), testosterone, finasteride, dithiothreitol (DTT), sucrose, MgCl_2_, nicotinamide adenine dinucleotide phosphate (NADPH), Tris-HCl buffer, and dichloromethane (DCM) were obtained from Sigma-Aldrich (St Louis, MO, USA). RNeasy Mini Kit was purchased from Qiagen (Hilden, Germany), and the catalase activity assay kit was obtained from Abcam (Cambridge, MA, USA).

### 4.2. 5α-Reductase Inhibitory Activity

Male Sprague Dawley rats at 7 weeks of age were prepared and anesthetized using ethyl ether. The whole liver of rat was extracted and lysed with STM buffer (270 mM sucrose, 10 mM Tris-HCl (pH 7.5), 1 mM MgCl_2_) to obtain the enzyme solution. The reaction was initiated by adding phosphate buffer (pH 6.5), 500 μM testosterone, and 770 μM NADPH to the prepared enzyme solution that had been treated with 125, 250, and 500 μg/mL of WHS or 125, 250, and 500 μM of hydrangenol. To terminate the reaction, DCM was added, followed by centrifugation at 2000 rpm for 10 min. The DCM layer was separated, concentrated, dissolved in 50% methanol, and quantified using HPLC. Inhibitory activity of 5α-reductase was calculated according to the following formula: Inhibitory activity (%) = [1 − (area value of control-area value of blank)/(area value of test sample − area value of blank)] × 100.

### 4.3. Cell Culture

Human follicle DPCs were obtained from PromoCell (Heidelberg, Germany), while HaCaT keratinocytes were purchased from the American Type Culture Collection (ATCC; Manassas, VA, USA). The DPCs were cultured in the DPC growth medium with the supplement mix and 1% antibiotic–antimycotic. HaCaT keratinocytes were cultured in DMEM supplemented with 10% FBS and 1% antibiotic–antimycotic. Both cells were maintained in a humidified atmosphere at 37 °C with 5% carbon dioxide.

### 4.4. MTT Assay

A 3-(4,5-dimethylthiazol-2-yl)-2,5-diphenyltetrazolium bromide (MTT) assay was employed to determine cell viability. DPCs were seeded in a 96-well plate at a density of 4 × 10^3^ cells/well. Following 24 h incubation, DPCs were treated with WHS (1.56–100 μg/mL) or hydrangenol 1.56–100 μM) dose-dependently for an additional 24 h incubation. Formazan crystals formed by treating with 0.5 mg/mL MTT were dissolved with DMSO. Absorbance was then measured at 540 nm using an Epoch microplate reader (BioTek Instruments, Winooski, VT, USA).

### 4.5. DPC Spheroid (3D) Culture

To evaluate the size of DPC spheroids, cells were seeded at a density of 4 × 10^4^ cells/well in a 96-well, round bottom plate. Cells were incubated for 24 h to generate spheroids with/without various concentrations of WHS (25, 50, 100 μg/mL) or hydrangenol (6.25, 12.5, 25 μM). The diameters of spheroids were measured using phase-contrast microscopy.

### 4.6. RNA Extraction and Quantification Real-Time RT-PCR

DPCs were seeded in a 60 mm dish at a density of 2 × 10^5^ cells/dish, and HaCaT keratinocytes were seeded in a 6-well plate at a density of 3 × 10^4^ cells/well; both were cultured for 24 h. Then, WHS (25, 50, 100 μg/mL) or hydrangenol (6.25, 12.5, 25 μM) was treated at different concentrations for 24 h. As a positive control, 20 μg/mL of finasteride was used. When required, cells were co-treated with 1 mM of H_2_O_2_ or 50 μM of DHT. Total cellular RNA was isolated using an RNA isolation kit (Qiagen, Hilden, Germany) according to the manufacturer’s protocol. After RNA isolation, the total RNA was treated with DNase I (Thermo Scientific, Waltham, MA, USA) for more than 30 min before cDNA synthesis was performed using reverse transcriptase (Toyobo, Osaka, Japan). Quantitative RT-PCR was carried out using a SYBR Green PCR Mix (Toyobo) with a CFX Duet Real-Time PCR System (Bio-Rad, Hercules, CA, USA) according to the manufacturer’s instructions. The primer sequences utilized for amplifying targeted genes were designed using Primer3 and the BLAST tool. The mRNA expression levels were determined employing the 2^−ΔΔCt^ method and normalized to the glyceraldehyde-3-phosphate dehydrogenase (GAPDH) housekeeping gene. The qPCR primer sequences are presented in Supplementary Table S1.

### 4.7. Catalase Activity

Catalase activity was determined using a commercially available kit (Abcam). DPCs were seeded in a 100 mm dish at a density of 5 × 10^5^ cells/dish. Upon reaching 70% confluency, the cells were treated with various concentrations of WHS (25, 50, and 100 μg/mL) or hydrangenol (6.25, 12.5, and 25 μM) for 20 h, and then 1 mM of H_2_O_2_ was added for 4 h to induce oxidative stress. Subsequently, the cells were harvested for catalase activity evaluation using a colorimetric assay.

### 4.8. Animal Experiments

C57BL/6 mice at 5 weeks of age were purchased from Orient Bio animal center (Seongnam, Republic of Korea) and housed in a controlled environment with an automatic 12 h light–dark cycle, a temperature of 24 °C ± 0.5 °C, a humidity level of 55–65%, and ad libitum access to food and water. All experiments were conducted according to the protocols approved by the Yonsei University Health System Institutional Animal Care and Use Committee (IACUC No. 2022-0062) and were performed in the specific pathogen-free (SPF) facility at the Avison Biomedical Research Center, Yonsei University of Medicine. The mice were acclimated to stable conditions for one week before experimentation. Following acclimatization, the mice were anesthetized with isoflurane, and then the hair of the dorsal area was removed using an animal clipper. Subsequently, depilatory cream (Niclean^®^, Il Dong Pharmaceutical, Seoul, Republic of Korea) was applied to the shaved area to completely remove residual hair. After 24 h for skin stabilization, mice with pinkish skin and visible hair regrowth were selected at 6 weeks of age. These mice were weighed and then divided into groups of 6 animals each and were orally administered WHS (75, 150, and 300 mg/kg/day) for 3 weeks. Body weight measurements were conducted to assess the impact of administration on body weight changes. On days 14 and 21 of the experiment, mice were euthanized using CO_2_ anesthesia.

### 4.9. Visual Analysis of Hair Growth

To assess hair growth, weekly photographs were taken using a scanner (Aura X Pro; CZUR, Shenzhen, China). Changes in hair growth on the dorsal area were observed visually, and the degree of hair growth was categorized into the following ranges: 0–19% (1–1.5 points), 20–39% (2–2.5 points), 40–59% (3–3.5 points), 60–79% (4–4.5 points), and 80–100% (5 points). The average score was calculated and determined by skilled researchers through blinded testing.

### 4.10. Measurement of Hair Length and Thickness

Total hair samples from each mouse dorsum were collected after the experiment concluded. These collected hairs were placed on glass slides and securely fixed. Subsequently, measurements were taken using an optical microscope (BX43F; Olympus, Tokyo, Japan). The hairs were magnified to 125 times their original size, and 10 strands per mouse were measured to calculate the average length and thickness.

### 4.11. Measurement of Hair Weight

Total hair samples from each mouse dorsum were collected after the experiment concluded. Hair samples with a surface area of 1 cm^2^ were weighed using a precision microbalance (Premium Microbalance (Cubis2); Satorious, Seoul, Republic of Korea).

### 4.12. Measurement of Hair Density

The skin tissue from mice in the second week of oral administration was extracted. The extracted tissues were fixed in 10% formalin for over 24 h, and paraffin blocks and paraffin-embedded slides were prepared. The deparaffinized tissues were stained with hematoxylin (104302; Merk, Kenilworth, NJ, USA) solution to stain the nuclei, followed by rinsing with running water. Subsequently, the tissues were stained with eosin (230251; Sigma) solution to stain the cytoplasm, followed by another round of rinsing with water. The dehydrated tissues were then mounted using a mounting solution. Hair follicle counts were determined on a 1 mm^2^ area of tissue, magnified 400× using an optical microscope (BX43F; Olympus) to measure the number of hair follicles in the epidermis and dermal tissues.

### 4.13. Generation of Skin Organoids and Assessment of Hair Formation

To evaluate the efficacy of promoting hair formation in ski organoids, WA25 cells (WiCell Research Institute, Madison, WI, USA) were seeded in 96-well U-bottom plates from day 3 to day 12, with different culture media required for differentiation at each time point. From day 12 onwards, the cells were transferred to Ultra-Low Attachment 24-well plates and cultured with media changes every three days until drug treatment. WHS was treated with the culture media every three days at concentrations of 100 μg/mL and 200 μg/mL from day 131 to day 170. The presence or absence of hair follicles in the skin organoids was then captured and analyzed using optical microscopy.

### 4.14. Statistical Analysis

The experimental data are expressed as mean ± standard deviation (SD). Statistical analyses comparing different groups were conducted using an independent *t*-test and one-way ANOVA. A significance level of *p* < 0.05 was considered statistically significant.

## 5. Conclusions

In summary, our research demonstrates that WHS shows significant potential as a hair growth promoter. WHS stimulates DPC growth and enhances the expression of key hair growth and health-related genes. It also exhibits anti-inflammatory and anti-androgenic effects. Both in vitro and in vivo experiments confirmed its efficacy in promoting hair growth, making WHS a promising nutraceutical for hair growth.

## Data Availability

Data are contained within the article or Supplementary Materials. The data presented in this study are available.

## Supplementary Materials

The following supporting information can be downloaded at: https://www.mdpi.com/article/10.3390/ijms251910370/s1.
