# Supplementary material for: Hair Growth-Promoting Effect of Hydrangea serrata (Thunb.) Ser. Extract and Its Active Component Hydrangenol: In Vitro and In Vivo Study
Source: Int J Mol Sci. 2024 Sep 26;25(19):10370. doi: 10.3390/ijms251910370 (PMC11477035; doi:10.3390/ijms251910370)
Supplement: Supplementary file 1 [file ijms-25-10370-s001.zip › (Supplementary Figures).pdf]

[Supplementary Figures]

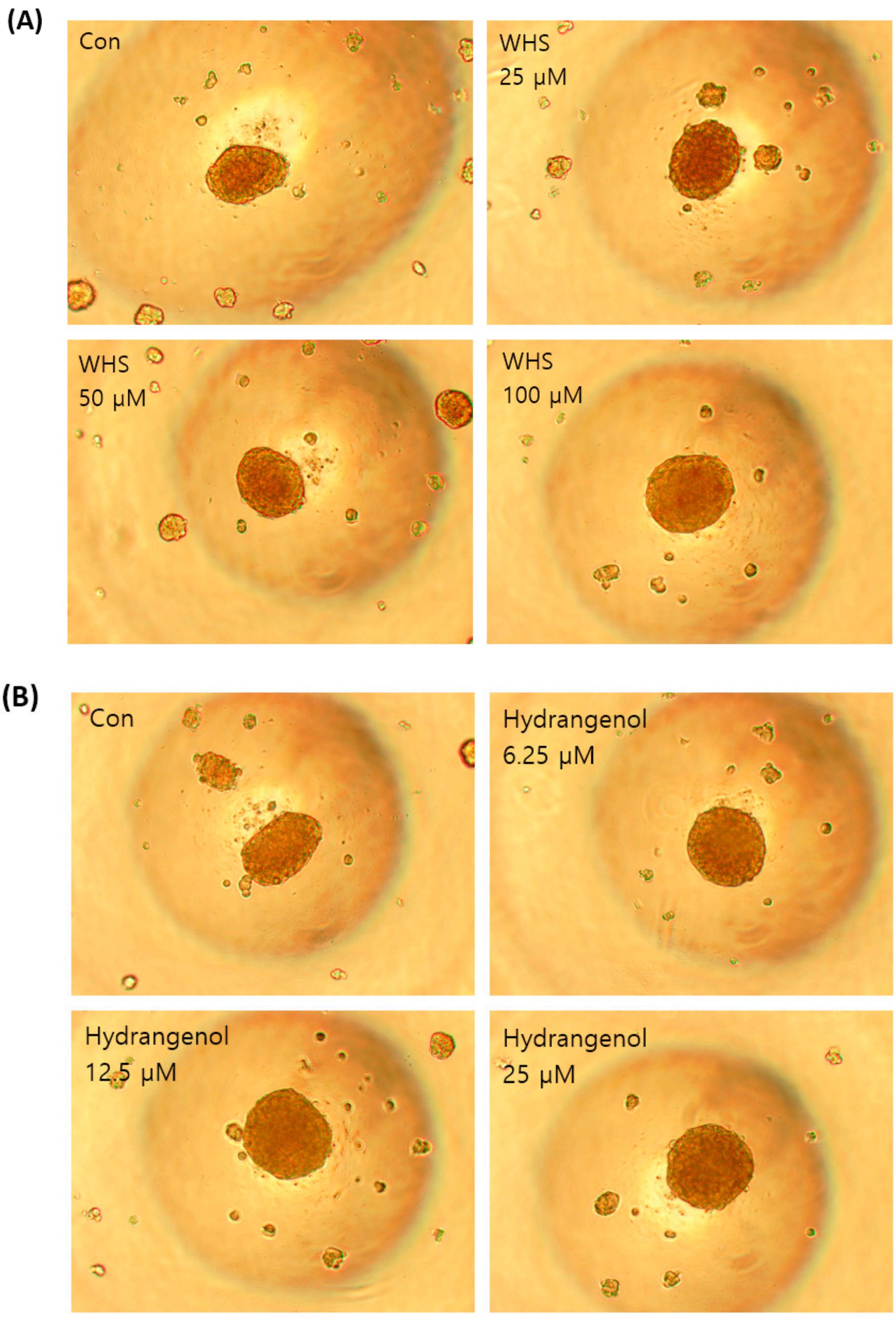

Figure S1. The size of DPC spheroids is quantified using phase contrast microscopy. (A) Representative images of DPC spheroids treated with WHS and (B) hydrangenol. WHS; water extract of *Hydrangea serrata* leaves.

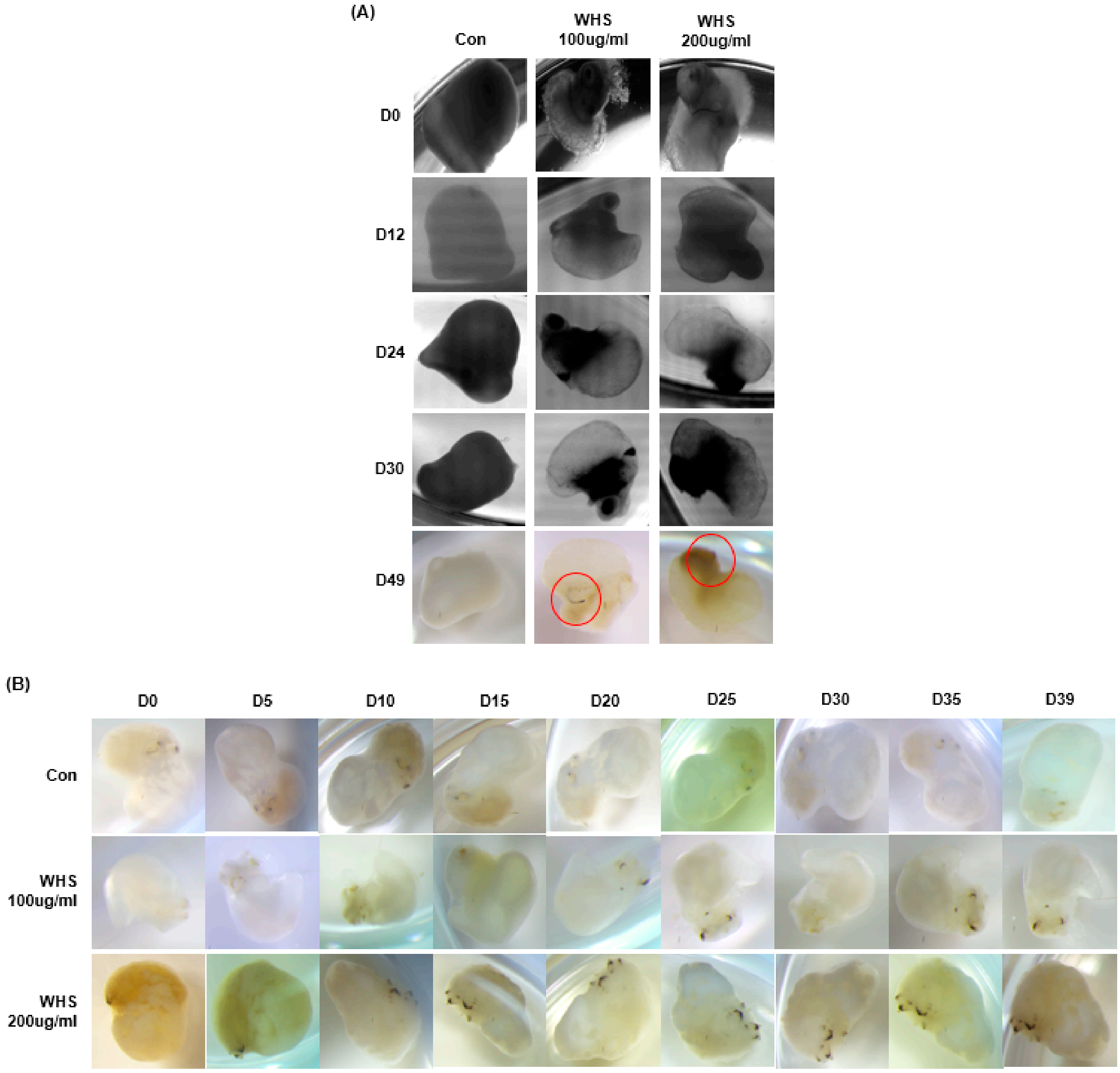

Figure S2. Effect of promoting hair formation by WHS in skin organoids. (A) Representative images of hair formation in WA25 skin organoids. Between days 82-131, hair formation was observed in both 100 μg/ml and 200 μg/ml of WHS-treated group, whereas no hair formation occurred in the non-treated condition. (B) Between days 131-170, hair formation was observed under all conditions; however it was particularly enhanced in the WHS treatment group. WHS; water extract of *Hydrangea serrata* leaves.

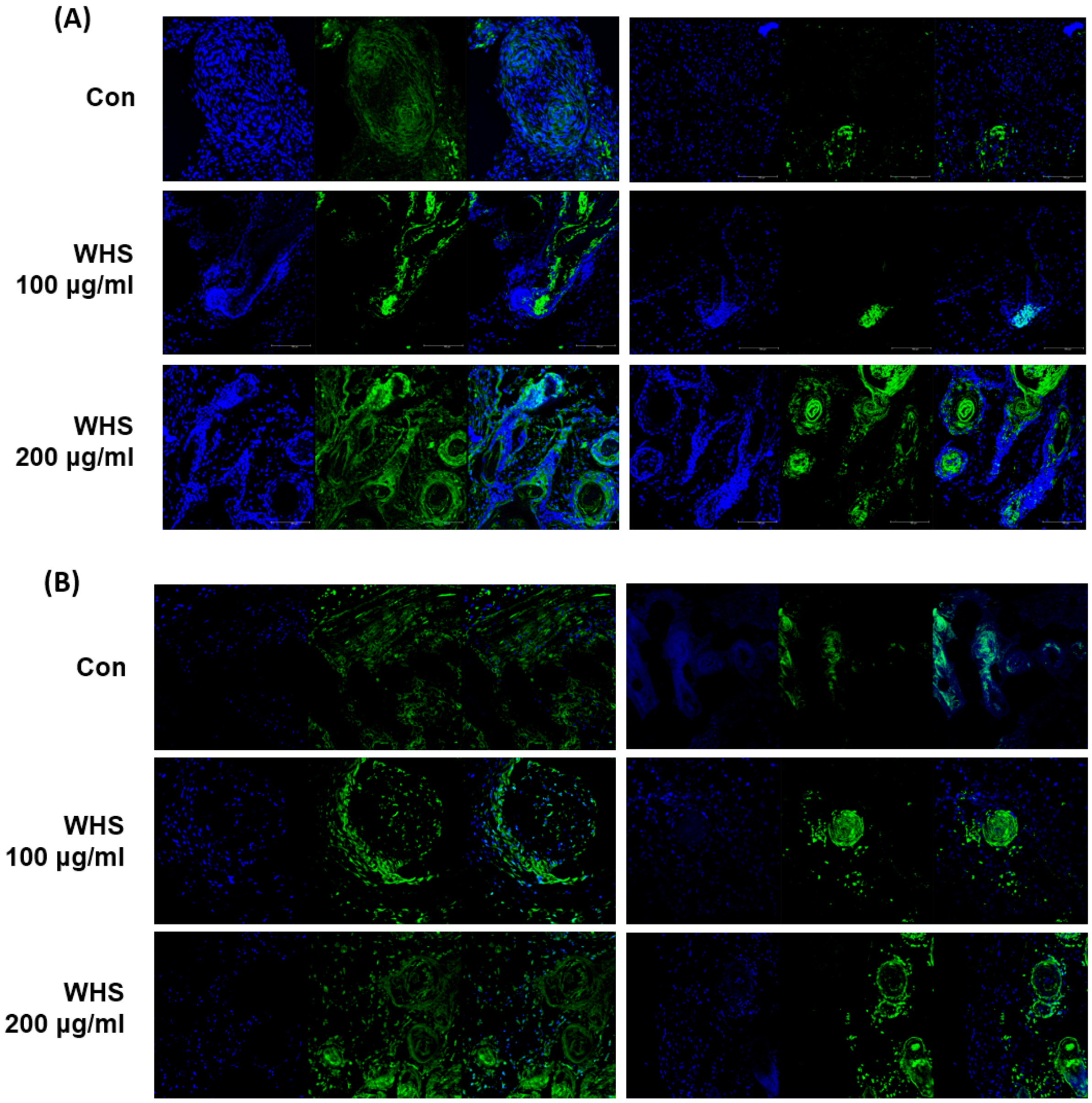

Figure S3. Effect of inducing hair follicle markers by WHS in skin organoids. (A) Representative immunostaining images for markers of basal layer (CK5) and hair follicle bulge (NFATC1) after day 131 and (B) after day 170. The basal layer marker CK5 was highly expressed under all conditions. The hair follicle bulge marker NFATC1 was detected at 100 µg/ml of WHS-treated group and showed increased expression at the higher concentration of 200 µg/ml. Nuclei are stained with DAPI. These results were consistent with the data in Fig S1. WHS; water extract of *Hydrangea serrata* leaves.

(A)

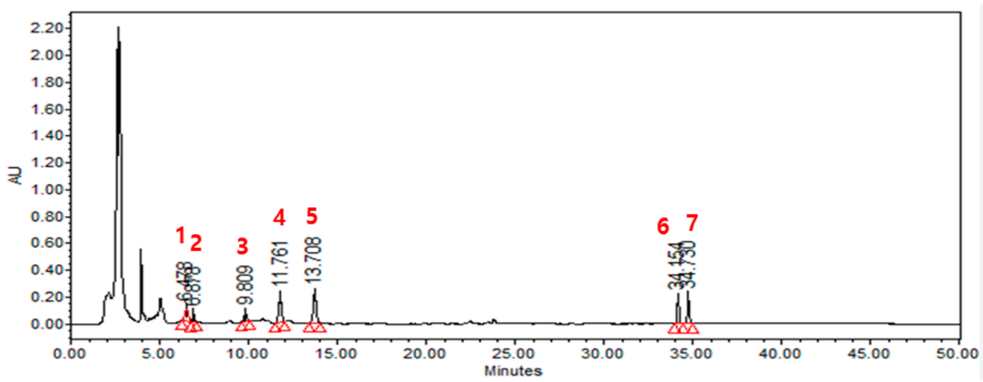

(B)

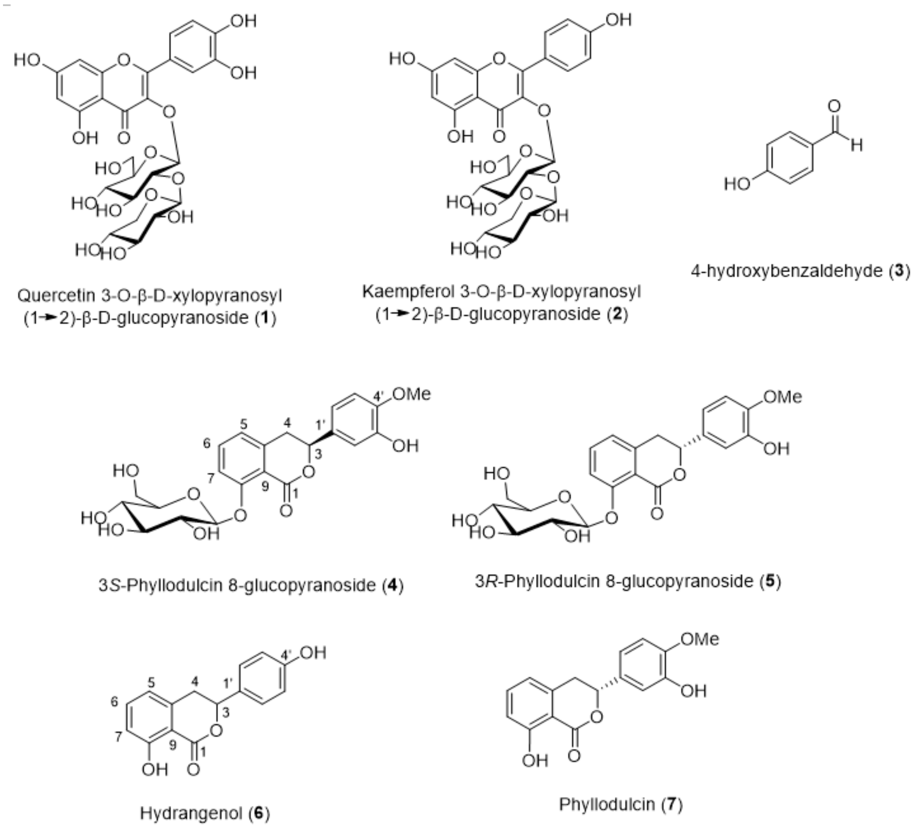

No	Compound	RT (min)	Contents (mg/g)
1	Quercetin 3-O-β-xylopyranosyl(1→2)-β-D-glucopyranoside	6.478	6.36
2	Kaempferol 3-O-β-xylopyranosyl(1→2)-β-D-glucopyranoside	6.878	3.77
3	4-Hydroxybenzaldehyde	9.809	4.24
4	3*S*-Phyllodulcin 8-glucoside	11.761	8.91
5	3*R*-Phyllodulcin 8-glucoside	13.708	10.47
6	Hydrangenol	34.154	6.80
7	Phyllodulcin	34.73	6.84

<HPLC analysis conditions>
- Column : Luna C18 (5 µm, 250 × 4.6 mm, Phenomenex)
- Detector : Waters 2998 PDA (228 nm)
- Pump : Waters 1525
- Oven temperature : 30℃
- Flow rate : 1 mL/min
- Solvent system : Gradient system

Time (min)	% A (ACN)	% B (H_2O)
**Initial**	20	80
**15**	25	75
**30**	50	50
**40**	100	0
**50**	20	80

Figure S4. The high performance liquid chromatography (HPLC) chromatogram used to measure the compounds of WHS. (A) HPLC profile of seven compounds isolated from WHS and (B) chemical structures and proportions of each compound. WHS; water extract of *Hydrangea serrata* leaves.
