# Supplementary material for: Hair Growth-Promoting Effect of Hydrangea serrata (Thunb.) Ser. Extract and Its Active Component Hydrangenol: In Vitro and In Vivo Study
Source: Int J Mol Sci. 2024 Sep 26;25(19):10370. doi: 10.3390/ijms251910370 (PMC11477035; doi:10.3390/ijms251910370)
Supplement: Supplementary file 1 [file ijms-25-10370-s001.zip › (Supplementary Method).pdf]

[Supplementary Materials and Methods]

Table S1. Primer sequences used for quantitative polymerase chain reaction (qPCR)

Gene name	Primer sequences (5'→3')
Keratinocyte growth factor (*KGF*)	F: CCTTCTGCCTGTTGATTTATGG<br>R: AGTTGCTGTGACGCTGTTTG
Fibroblast growth factor 10 (*FGF10*)	F: GCATTCTGCCTTCATCCCTTTC<br>R: TAGCACACGGGCACTCATAC
Transforming growth factor-β1 (*TGF-β1*)	F: GGTGGAATACGGCAACAAAATC<br>R: TGCTGCTCCACTTTTAACTTGA
Keratin 31 (*K31*)	F: TGCGCTAGAACCTAGGGAATG<br>R: GAGCAGGACAGTCTGGAGTAG
Keratin 85 (*K85*)	F: CAGACTCTGCTCAGCCTCACAC<br>R: CGGAAGCCACCTACAGCTATCC
Ceramide synthase 3 (*CerS3*)	F: CCAGGCTGAAGAAATTCCAG<br>R: AACGCAATTCCAGCAACAGT
Tumor necrosis factor-α (*TNF-α*)	F: CAGGCGGTGCCTATGTCTC<br>R: CGATCACCCCGAAGTTCAGTAG
Androgen receptor (*AR*)	F: CCAGCAGAAATGATTGCAC<br>R: ATTACCAAGTTTCTTCAGCTTC
Dickkopf-1 (*DKK-1*)	F: GCGGGAATAAGTACCAGAC<br>R: CGCAGTACTCATCAGTGCC

1.1. Immunofluorescence Staining

To investigate the hair follicle markers in skin organoids, immunofluorescence analysis was conducted. Whole-mount immunostaining was performed, involving fixation of samples in 4% (v/v) paraformaldehyde (PFA), permeabilization, incubation with primary antibodies, subsequent incubation with fluorescently labelled secondary antibodies, re-fixation in 4% (v/v) PFA and clearing. Analysis was carried out using a confocal microscope (Leica TCSSP5 II; Leica, Wetzlar, Germany). The expression of follicle markers was evaluated under non-treated conditions and conditions treated with WHS. The information regarding the antibodies used for analysis is listed in Table S2.

[Table S2] Antibodies list

Antibody	Vendor	Cat No.	Markers for:
DAPI	Thermo Fisher	H-1399	Nucleus
Cytokeratin 5 Monoclonal Antibody (2C2)	Thermo Fisher	MA5-17057	Basal layer of skin
NFATC1 Monoclonal Antibody (7A6)	Invitrogen	MA3-024	Hair follicle bulge

Goat Anti-Mouse IgG H&L (Alexa Fluor®488)	Abcam	Ab150113	Secondary antibodies
